# Preface to Nitric Oxide Modulators in Health and Disease I

**DOI:** 10.3390/molecules27206820

**Published:** 2022-10-12

**Authors:** Cristina Maccallini, Rosa Amoroso

**Affiliations:** Department of Pharmacy, University “G. d’Annunzio” of Chieti-Pescara, Via dei Vestini, 31, 66100 Chieti, Italy

## 1. Introduction

Nitric oxide (NO) is a small free radical molecule biosynthesized by nitric oxide synthases (NOS), a family of oxidoreductases responsible for the conversion of the natural substrate L-arginine into L-citrulline and NO. The latter plays essential biological roles in the human body, taking part in the innate immune response and regulating vascular homeostasis as well as both the central and peripheral nervous system functions [1].

However, there are many different pathological conditions related to imbalanced NO bioavailability, in which either the under- or the overexpression of NOS occurs. Therefore, research on therapeutic interventions aiming to restore the proper NO production and bioavailability is an attracting research field. Based on the specific disease and the effective bioavailable NO levels, there are two possible options to modulate the nitrergic system, i.e., the administration of NO donors, which generate in situ NO, or NOS inhibitors, which modulate the improper overactivity of the NOS. Both these medicinal approaches show pros and cons, in a continuous updating of scientific results and potential applications.

The present Special Issue, having collected four original research articles and four reviews, aims to give some new insights into the modulation of NO signaling as a valuable therapeutic approach for the treatment of pathological conditions associated with unsafe NO bioavailability. In the next section, an overview of the collected papers is presented.

## 2. Special Issue Overview

Nitric oxide is a well-established neuromodulator, with a role in the modulation of circadian entrainment [2]. Plano S. A. et al. demonstrated that the pharmacological administration of Angeli’s Salt, a diazeniumdiolate-based nitroxyl (HNO) donor, is able to modulate photic entrainment in the hamster suprachiasmatic nuclei [3]. Therefore, the authors give evidence on the capability of HNO, which is a redox-dependent form of NO, to act as a noncanonical messenger in the control of the circadian phase.

NO-releasing drugs are useful double agents targeting specific proteins, while donating, through a synergistic method of action, controlled amounts of NO in the tissue environment. Prostanit, i.e., PGE1 dinitroglycerol ester, is a NO-donating PGE1 derivative with vasodilatory and antiplatelet properties. Given its potential therapeutic effect in the management of peripheral arterial disease (PAD), in their study, Shestakova K. M. et al. assessed both in vitro and in vivo the pharmacokinetic properties and metabolic activation of Prostanit [4]. Interestingly, the authors found a double-phase drug activation, which allows not only the direct release of NO but also the stimulation of NOS activity. Moreover, the authors performed a pharmacometabolomic study, finally suggesting that Prostanit may deserve further investigation in PAD models.

S-nitrosothiols (SNO) are thiol-NO adducts involved in many different physiological functions, and they can be also considered as tissue NO storage. However, the presence of very high levels of SNO levels in proteins could be associated with several disease states [5]. Neidigh N. et al. used a non-invasive analytical method to measure S-nitrosothiols in vivo, by detecting NO released from the photolysis of biologically relevant SNO [6]. This method could be useful to evaluate SNO in a variety of tissues in which they are known to regulate cell signaling.

The dysregulation of NOS, and in particular of the inducible isoform, is recognized as a critical factor in the development of different diseases, including cancer [7]. Therefore, inhibitors of iNOS could be a useful in the treatment of all the conditions related to the excessive biosynthesis of NO, and many different iNOS inhibitors have been studied to date [8,9,10]. Since monocytes, as innate effector cells, also have a central role in the progression of inflammation in different diseases, such as autoimmune diseases, cancer, and atherosclerosis, Gallorini M. et al. investigated if the inhibition of iNOS by means of new acetamidine-based compounds could affect monocytes’ responses to pro-inflammatory stimuli [11]. The obtained results point to the immunomodulatory effects of the studied molecules.

The development of new NOS inhibitors could also be useful in the management of pain syndromes. As reviewed by Shnayder N.A. et al., in patients with peripheral chronical pain syndromes, an association can be found between single-nucleotide variants of *NOS1*, *NOS2*, and *NOS3* genes encoding nNOS, iNOS, and eNOS and acute and chronic peripheral pain [12]. Therefore, the administration of the appropriate NOS inhibitor could be considered as a new personalized pharmacotherapy strategy. Further, Shnayder N.A. et al. also summed up recent findings on single-nucleotide variants of *NOS1*, *NOS2*, *NOS3* genes involved in the development of tension-type headache (TTH) associated with arterial hypertension (AH) [13]. Although the results of the studies discussed in this review are contradictory, a potential therapeutic effect of iNOS inhibitors and NO scavengers in patients affected by essential AH and TTH is suggested.

In their review article, A.M. Pourbagher-Shahri et al. discuss how the critical changes in NO bioavailability are related to the human aging process and to the development of age-related diseases, including cardiovascular, neurological, reproductive, skin, renal, thyroid, muscle, and sleep disorders. An overview of the chemical and natural agents that can enhance both eNOS and nNOS expression and inhibit iNOS is given, suggesting their potential use to ameliorate the mentioned pathological conditions [14].

Starting from well-documented relationships between NO bioavailability and schizophrenia development, in their review, Zoupa E. et al. critically assessed advances in the research of the NO-donor sodium nitroprusside for the therapy of schizophrenia, discussing its potential superiority over currently used neuroleptics [15].

## 3. Conclusions

The articles collected in this Special Issue offer new perspectives in the application of NO-related knowledge. New therapeutic applications of NO-donating drugs, as well as of NOS inhibitors, are described and discussed, and a critical revision of the literature on the relationships between NO bioavailability and selected diseases occurrence is offered.
